# A semi-parametric statistical model for integrating gene expression profiles across different platforms

**DOI:** 10.1186/s12859-015-0847-y

**Published:** 2016-01-11

**Authors:** Yafei Lyu, Qunhua Li

**Affiliations:** The Huck Institute of Life Science, Pennsylvania State University, University Park, PA 16802 USA; Department of Statistics, Pennsylvania State University, University Park, PA 16802 USA

**Keywords:** Data integration, Gene expression, Copula, Mixture model, Rank, Meta-analysis

## Abstract

**Background:**

Determining differentially expressed genes (DEGs) between biological samples is the key to understand how genotype gives rise to phenotype. RNA-seq and microarray are two main technologies for profiling gene expression levels. However, considerable discrepancy has been found between DEGs detected using the two technologies. Integration data across these two platforms has the potential to improve the power and reliability of DEG detection.

**Methods:**

We propose a rank-based semi-parametric model to determine DEGs using information across different sources and apply it to the integration of RNA-seq and microarray data. By incorporating both the significance of differential expression and the consistency across platforms, our method effectively detects DEGs with moderate but consistent signals. We demonstrate the effectiveness of our method using simulation studies, MAQC/SEQC data and a synthetic microRNA dataset.

**Conclusions:**

Our integration method is not only robust to noise and heterogeneity in the data, but also adaptive to the structure of data. In our simulations and real data studies, our approach shows a higher discriminate power and identifies more biologically relevant DEGs than eBayes, DEseq and some commonly used meta-analysis methods.

**Electronic supplementary material:**

The online version of this article (doi:10.1186/s12859-015-0847-y) contains supplementary material, which is available to authorized users.

## Background

Detection of differentially expressed genes (DEGs) between biological samples is the key to understand how genotype gives rise to phenotype. With the rapid accumulation of consortium studies (e.g. ENCODE [1]) and public repositories (e.g. NCBI GEO [2]), a large number of RNA-seq and microarray data collected on similar samples from different sources have been made publicly available. Such collections make it possible to integrate similar studies from different sources and platforms in transcriptome analyses, potentially increasing statistical power and reliability in DEGs detection [3–6], while decreasing the cost of the analyses.

Despite these well-known benefits, combining gene expression data from different sources involves many intricate issues. For example, if data are collected from different platforms, the scales of measurements on individual studies may not be comparable. Even though many normalization methods have been developed, normalization across platforms still remains a challenge [7–10]. Furthermore, heterogeneity is often present in studies from different sources. Lab effects often are still retained among datasets produced by different laboratories even after normalization [11].

A convenient way to handle heterogeneity and noise in the data is to use rank-based approach, since rank is robust to outliers and is always comparable across platforms. It has been shown that ranking fold changes of differential gene expression produces better agreement of DEG lists across labs and platforms than using p-values in microarray gene expression studies [12]. Recently, several rank-based data integration methods have been developed, for example, RankProd [13], RankSum [13], product of ranks, [14] and sum of ranks [14]. They have been found effective in overcoming the heterogeneity among datasets. However, these methods typically are nonparametric methods with fixed rules for combining studies, such as, computing convolution of (transformed) ranks of fold change, hence are not adaptive to the structure of the data.

In this work, we develop a rank-based semi-parametric statistical method to integrate gene expression profiles from different sources. Our model emphasizes the biological intuition that true signals from samples measuring the same biological mechanism should have concordant signals. It builds in a strong preference for concordance of differential directionality and significance across sources, such that the genes that have moderate but consistent signals across studies can be effectively detected. Unlike meta-analysis methods based on fixed rules, our method explicitly models the structure of the data through a copula mixture model, making it both adaptive to the data and robust to noise.

We illustrate our method in the integration of microarray and RNA-seq data for DEG detection. Microarray has been the major experimental platform for gene expression study since mid-1990’s [15, 16]. Despite its huge success, it is known to suffer from some limitations such as reliance on existing knowledge of transcript sequences, high background noise, and limited dynamic range of detection. Recently, RNA-Seq has emerged as a new experimental platform for transcriptome profiling and has been flourishing since then. Though it is believed that RNA-seq overcomes the major limitations of microarray [17], RNA-seq still demonstrates excessive variability [18], especially when sequencing depth is low or the gene expression level is low [1, 5]. Considerable differences have been reported between the DEGs detected in these two platforms [19–24]. In addition, due to cost constraints, many RNA-seq experiments nowadays still have no replicates, which limits the power and reliability of its inference. When both microarray and RNA-seq data are available for the same sample, it is natural to investigate whether integrating the data from these two platforms will combine the strengths of the platforms and improve the reliability of DEG identification.

We apply our method to microarray and RNA-seq data from the Microarray Quality Control (MAQC) [12] and Sequencing Quality Control (SEQC) [3] projects, as well as a synthetic microRNA dataset [25]. Our results show that our method substantially improved the accuracy for detecting DEGs.

## Methods

### Statistical model for gene expression profiles across platforms

Our goal is to develop a data integration method that is both robust to noise and heterogeneity in the data, and adaptive to the structure of data. We develop our method based on a copula mixture model in Li et al (2011) [26]. This model is originally developed for assessing the reproducibility of rank orders between two rank lists from high-throughput experiments. It has been successfully applied to the analysis of ChIP-seq data in ENCODE for comparing peak callers, identifying suboptimal experiments, and determining reporting thresholds for ChIP-seq peaks [27, 28].

Though this method was originally proposed for assessing reproducibility, it can also be viewed as a semi-parametric aggregation method that combines rank lists from different studies. The signals that are consistent across studies are weighed more favorably than those with similar significance but inconsistent across studies. However, this model only clusters the entries into two groups, with the top - ranked ones as interesting signals and the bottom - ranked ones as noise; whereas, for gene expression studies, DEGs reside on both ends of the rank lists, and both ends would be of interest.

Here we extend this model to the context of gene expression studies. We assume that the sample consists of non-DE, up-regulated, and down-regulated DE genes. We use the level of differential expression (e.g. fold change) as our data, such that the up-regulated, down-regulated, and non-DE genes are concentrated on the top, bottom, and middle part of the rank lists, respectively. For simplicity of discussion, we focus on the case of two studies in what follows, and provides the extension to the case with more studies in Additional file 1.

Suppose the level of differential expression for gene *i* on two studies are (*x*_*i*,1_, *x*_*i*,2_), we assume that *X*_*j*_ = (*x*_1,*j*_, *x*_2,*j*_, …, *x*_*n*,*j*_), *j* = 1, 2 is an independent and identically distributed sample with CDF *F*_*j*_, where *F*_*j*_ is unknown and can vary across studies. Let *K*_*i*_ denote whether the *i*^th^ gene is non-DE (*K*_*i*_ = 0), up-regulated (*K*_*i*_ = 1) or down-regulated (*K*_*i*_ = 2), and let *π*_0_, *π*_1_ and *π*_2_ = 1 − *π*_0_ − *π*_1_ denote the corresponding proportions.

Because differentially expressed genes are expected to be concordant in both the direction of differentiation and the level of significance across studies, we expect the differential expression level of a gene to be positively correlated across studies for DEGs but not for non-DEGs. To model this dependence structure, we assume that, given *K*_*i*_ = *k*, the dependence across studies for the genes in the *k*^th^ component is induced by a latent bivariate Gaussian random variable, ***z***_***k***_ = (*z*_*i*,1,_*z*_*i*,2_)|*K*_*i*_ = *k*. The correlation coefficient between the two studies *ρ*_*k*_ is positive for ***z***_1_ and ***z***_2_, and 0 for ***z***_0_. Though the marginal distribution of observed differential expression level, *F*_*j*_, may be different across studies, it is natural to assume *z*_*i*,1_|*K*_*i*_ = *k* and *z*_*i*,2_|*K*_*i*_ = *k* have the same marginal distributions, as different studies are assumed to measure the same underlying biological process. To reflect up- and down-regulation, we assume ***z***_1_ has a higher mean than ***z***_0_, and ***z***_0_ has a higher mean than ***z***_2_. Finally, as the scales of the marginal distributions are unknown, only the difference in means between two latent variables and the ratio of their variances can be identified, but not their actual means and variances. Thus, we set ***z***_0_ to have mean 0 and variance 1. Putting the above together, the model that generates the dependence structure is

1 $$ \left.\left(\begin{array}{c}\hfill {z}_{i,1}\hfill \\ {}\hfill {z}_{i,2}\hfill \end{array}\right)\right|{K}_i=k\sim {h}_k=N\left(\left(\begin{array}{c}\hfill {\mu}_k\hfill \\ {}\hfill {\mu}_k\hfill \end{array}\right),\left(\begin{array}{cc}\hfill {\sigma}_k^2\hfill & \hfill {\rho}_k{\sigma}_k^2\hfill \\ {}\hfill {\rho}_k{\sigma}_k^2\hfill & \hfill {\sigma}_k^2\hfill \end{array}\right)\right) $$

where *μ*_0_ = 0, *σ*_0_^2^ = 1, *ρ*_0_ = 0, *μ*_1_ > 0 > *μ*_2_, 1 > *ρ*_*k*_ > 0 for *k* = 1, 2. Let ${u}_{i,j}\equiv G\left({z}_{i,j}\right)={\displaystyle {\sum}_{k=0}^2}{\pi}_k\Phi \left(\left({z}_{i,j}-{\mu}_k\right)/{\sigma}_k\right)$, where Φ(·) is the CDF of the standard normal distribution. Then our actual observations *x*_*i*,*j*_ are

$$ {x}_{i,j}={F}_j^{-1}\left({u}_{i,j}\right). $$

Our model can be parameterized by *θ* = (*π*_0_, *π*_1_, *π*_2_, *μ*_1_, *μ*_2_, *σ*_1_, *σ*_1_, *ρ*_1_, *ρ*_2_) and (*F*_1_, *F*_2_), where *F*_1_ and *F*_2_ will be substituted by the empirical distributions if they are unknown. Thus it is scale-free. The corresponding mixture likelihood for the data is

$$ L\left(\theta \right)={\displaystyle \prod_{i=1}^n\left[{\displaystyle \sum_{k=0}^2{\pi}_k{h}_k\left({G}^{-1}\left({F}_1\left({x}_{i,1}\right)\right),{G}^{-1}\left({F}_2\left({x}_{i,2}\right)\right)\right)}\right]}, $$

where *h*_*k*_ is the bivariate normal density function with parameters *μ*_*k*_, *σ*_*k*_^2^ and *ρ*_*k*_. The parameters *θ* can be estimated by maximizing the mixture likelihood using an estimation procedure similar to Li et al [26], with adaptation to three components. The detailed algorithm is provided in Additional file 1, Section 1.

### Determination of differential expression

Given the parameter *θ*, the posterior probability that a gene *i* is in the *k*^th^ group can be computed as

$$ {p}_k\left({x}_{i,1},{x}_{i,2}\right)=\frac{\pi_k{h}_k\left({G}^{-1}\left({F}_1\left({x}_{i,1}\right)\right),{G}^{-1}\left({F}_2\left({x}_{i,2}\right)\right)\right)}{{\displaystyle \sum_{k=0,1,2}{\pi}_k{h}_k\left({G}^{-1}\left({F}_1\left({x}_{i,1}\right)\right),{G}^{-1}\left({F}_2\left({x}_{i,2}\right)\right)\right)}} $$

The classification of a gene is determined by the component that possesses the highest posterior probability. To determine the cutoff for DEGs, we follow a selection procedure similar to the selection based on the IDR (irreproducible discovery rate) criterion in [26] with adaptation to three components, as follows:

1. Rank genes from low to high by *p*_0_(*x*_*i*,1_, *x*_*i*,2_), *i* = 1, … *n*
2. For the *i*^th^ ordered gene, compute $Pr\left( nonDG\Big|l\in {I}_i\right)={\displaystyle \sum_{l\in {I}_i}}{p}_0\left({x}_{l,1},\ {x}_{l,2}\right)/i$, where *I*_*i*_ = {(*x*_*l*,1_, *x*_*l*,2_) : *p*_0_(*x*_*l*,1_, *x*_*l*,2_) < *p*_0_(*x*_(*i*),1_, *x*_(*i*),2_)}.
3. For a desired control level *α*, let *i*_*max*_ = *max*{*i* : *Pr*(*nonDEG*|*l* ∈ *I*_*i*_) < *α*}, then differentially expressed genes can be selected by selecting the genes (*x*_(*l*),1_, *x*_(*l*),2_) with *l* = 1, …, *i*_*max*_. This set of genes will have an expected rate of nonDEG discoveries no greater than *α*.

*Pr*(*nonDEG*|*l* ∈ *I*_*i*_) represents the expected proportion of nonDEG genes in the claimed DEGs, when the cutoff is set at the *i* th ordered gene.

### Properties of this model

This model has several desirable properties as a data integration method. First, by modelling the two DEG components with positive correlation, the model builds in a strong preference for common directionality of significance across studies while not requiring the differential direction is known *a priori*. This is desirable, as the genes with concordant differentiation across studies are more likely to be real than the discordant ones. Second, the three-component clustering framework allows our method to estimate the two tails adaptively according to the data. When the proportions of up- and down-regulation are unequal, the asymmetry can be reflected in the clusters. In contrast, commonly-used meta-analysis methods, such as Fisher [29], Stouffer [30], and RankProd [13], implicitly assume that the rejection regions are symmetric on both sides, thus making it likely to lose power when asymmetry is present. Third, this model is scale-free, thus it is suitable for combining measurements on different scales or platforms. In our simulation and real data analyses, we will compare with both single-platform DEG detection methods and several commonly-used meta-analysis methods to illustrate the effectiveness of our method.

## Results and discussion

### Simulation studies with violation of model assumptions

We first examine the performance of our approach using a simple simulation study. In this simulation, the log fold changes are generated from a model similar to our model but with some violation of model assumptions. Our goal is to assess the robustness of our method against violation of model assumptions and to compare with commonly-used meta-analysis methods in this scenario.

Here we use model (1) as the basis to simulate the log fold changes on the two platforms. However, instead of assuming that the log fold change of all the up-regulated (or down-regulated) genes have the same distribution as in our model, we allow them to have different means and correlations, by letting *μ*_*k*_ and *ρ*_*k*_ (*k* = 1, 2) drawn from uniform distributions. This setting is more flexible than model (1) and introduces mild violation to our model assumptions. Here we choose *μ*_1_ ~ *unif*(0.58, 1.58), *μ*_2_ ~ *unif*(−1.58, 0.58), *ρ*_1_ ~ *unif*(0.80, 0.88) and *ρ*_2_ ~ *unif*(0.80, 0.88). For each gene, we obtain a p-value on each platform from a two-sided z-test for *H*_0_ : *μ* = 0 vs *H*_1_ : *μ* ≠ 0.

To compare our method with commonly-used meta-analysis methods, we combine the p-values using Fisher’s method and Stouffer’s method, and combine the log fold changes using our method and RankProd. RankProd is a non-parametric statistic that detects items that are consistently highly ranked in a number of lists [13]. Denote *r*_*i*,*j*_ as the rank of the fold change of the *i*^th^ gene in the *j*^th^ platform and *n*_*j*_ as the total number of genes in the *j*^th^ platform, RankProd is computed as $R{P}_{i,j}={\displaystyle \prod_{j=1,2}}\frac{r_{i,j}}{n_j}$. A small value of RP indicates that a gene is consistently highly ranked across platforms.

We evaluate the performance in four scenarios: (S1) data with same proportion of up- and down-regulated DEGs, (S2) data with different proportions of up- and down-regulated DEGs, (S3) data with a small proportion of DEGs, and (4) data with low inter-platform consistency. The parameter setting is shown in Additional file 1: Table S1. For each parameter setting, we simulate 100 data sets, each of which consists of two replicates with 5,000 genes on each replicate.

### Results of simulation studies with violation of model assumptions

**Fig. 1:**
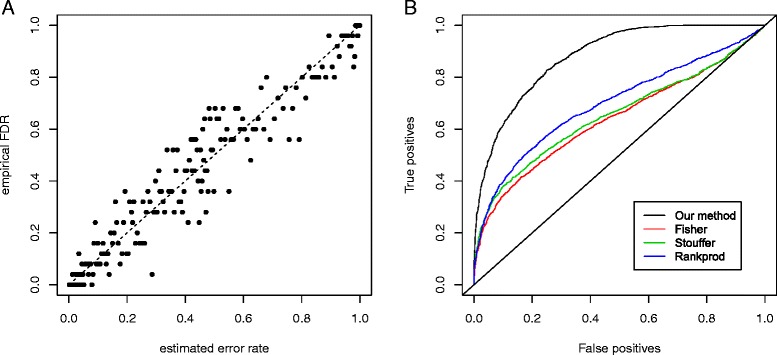
Calibration and comparison of discriminative power in the simulation with violation of model assumption. **a**. The estimated error rate is compared with the empirical FDR for the simulation setting S1. Results from other simulation settings are similar (Additional file 1: Figure S1). **b**. The percentage of correct and incorrect calls at various thresholds for our method, Fisher’s method, Stouffer’s method and RankProd, for the simulation setting S1. Results from other simulation settings are similar (Additional file 1: Figure S2)

In all simulations, our estimates for *μ*_*k*_ and *ρ*_*k*_ (*k* = 1, 2) are close to the means of the corresponding uniform distributions, and the other estimated parameters are close to the true parameters (Additional file 1: Table S1). As a guide for the selection of the signals, the error rate of non-DEG discoveries estimated from our method should be well calibrated. To check the calibration, we compare the actual frequency of false calls (i.e. empirical FDR) with the estimated error rate, *Pr*(*nonDEG*|*l* ∈ *I*_*i*_). As shown in Fig. 1a and Additional file 1: Figure S1, our method is well-calibrated in all the scenarios. In addition, we also evaluate the trade-off between the numbers of correct and incorrect calls made at various thresholds for all methods. As shown in Fig. 1b and Additional file 1: Figure S2, our method shows the highest discriminative power among all the methods of comparison. These results indicate that our method is robust to mild violation of model assumptions.

### Real data-based simulation studies

In this simulation, we simulate RNA-seq data and microarray data based on a real data set, following [31] and [32], respectively, and compare the performance of our method with single-platform DEG identification methods and some commonly-used meta-analysis methods in this more realistic setting.

To identify DEGs from a single-platform, we use DEseq for RNA-seq [33] and eBayes for microarray [34]. DEseq is one of the most commonly-used tools for identifying differentially expressed transcripts in RNA-seq data. It models the read counts based on a negative binomial model, with variance and mean linked by a data-driven local regression, and infers the significance of differentiation using an approach analogous to the Fisher’s exact test. eBayes is a popular method for determining DEGs for microarray data. It estimates mean and variance of gene expression levels based on an empirical Bayes framework, and determines the significance of differentiation according to the empirical Bayes moderated *t*-statistics and their associated p-values. To compare our method with commonly-used meta-analysis methods, we combine the p-values from DEseq and eBayes using Fisher’s method and Stouffer’s method, and combine the fold changes using RankProd and our method.

In an attempt to simulate realistic data, we estimated the parameters for simulation from a real dataset in Marioni et al [23], which consists of microarray and RNA-seq measurements of the same biological samples from two cell types (kidney and liver), and then simulated the distribution of the gene expression levels on each platform based on the estimated parameters.

### Simulation procedure for real data-based simulation studies

Here we provide a brief description of the simulation procedure. A detailed description and parameter settings can be found in Additional file 1, Section 2. Briefly, our simulation procedure consists of three parts, namely, simulation of the distribution of RNA-seq data, simulation of the expression levels of microarray data, and coupling of RNA-seq and microarray data. The counts of RNA-seq data are simulated from a negative binomial model following Kvam et al [31], with the mean parameter based on an estimate from the RNA-seq measurements of the kidney/liver samples in [23] and the over dispersion parameter drawn from a gamma distribution following Hardcastle and Kelly [35]. The microarray data is simulated following Xiao et al [32], where both the gene expression levels and the log-fold changes are simulated based on the estimates from the microarray measurements of the kidney/liver samples in [23]. After obtaining the distributions of RNA-seq and microarray data, the expression level of a gene on each platform then is generated by sampling the same quantile from the corresponding distribution.

Here we evaluate the performance in several scenarios, in particular, the scenarios when two platforms have similar versus different data quality, when data quality is high versus low, and when the proportions of up- and down-regulated genes are equal versus unequal. For each scenario, three replicates are simulated under two conditions for each platform, with 10,000 genes for each replicate. Simulated expression levels then undergo the standard pre-processing procedure (Additional file 1, Section 3) prior to the application of DEG detection methods.

### Results of real data-based simulation studies

**Fig. 2:**
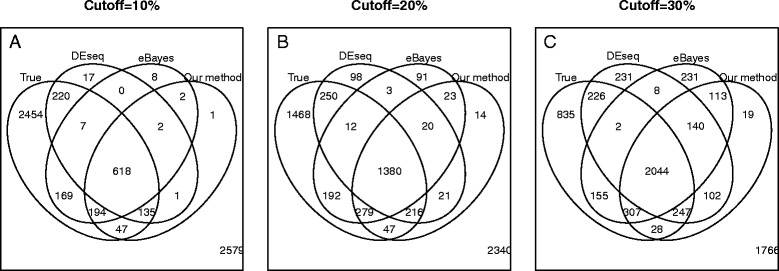
Venn diagram for the detected DEGs reported by different methods in the real data-based simulation. Expression levels of 10000 genes were simulated, among them 30 % genes are up regulated, 30 % genes are down regulated and 40 % genes are non-DE genes. After removing genes with low counts, 6454 genes remain. The Venn diagrams (from left to right) show the intersection between true DEGs and top 10 %, 20 % and 30 % genes ranked by DEseq, eBayes and our method, respectively. At 10 % cutoff, the detected true positives are 994/3844 = 25.8 % (our), 980/3844 = 25.4 % (DEseq) and 988/3844 = 25.7 % (eBayes), respectively. At 20 % cutoff, the detected true positive are 1922/3844 = 50.0 % (our), 1858/3844 = 48.3 % (DEseq) and 1863/3844 = 48.5 % (eBayes), respectively. At 30 % cutoff, the detected true positive are 2626/3844 = 68.3 % (our), 2508/3844 = 65.4 % (DEseq) and 2519/3844 = 65.9 % (eBayes)

**Fig. 3:**
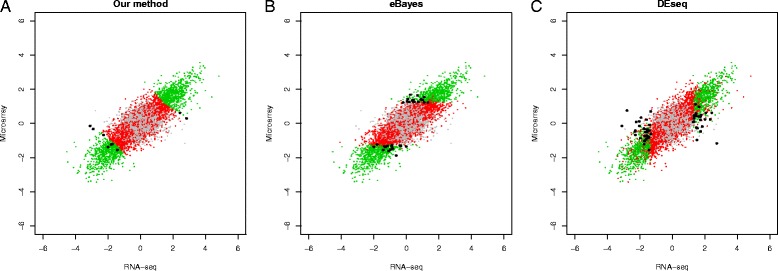
Comparison of decision boundaries in the real data-based simulation study. The log2 fold changes of gene expression levels are shown for genes in the simulation in Fig. 2. The genes that are ranked in the top 20 % by each method are deemed as DEGs. Correctly detected DE genes, false positive, false negative and true negatives from each method are shown in green, black, red and grey, respectively. The difference in decision boundaries shows that genes with moderate but consistent signals are more likely to be identified by our method

As the significance levels from different methods may not be directly comparable, we evaluate the accuracy of DEG identifications for the top 10 %, 20 % and 30 % genes ranked by each method. At all three cutoffs, our method identifies more true DEGs than eBayes and DEseq. For example, when the cutoff is at 30 %, our method identifies 2626/3844 = 68.3 % true DEGs, whereas eBayes and DEseq identify 2508/3844 = 65.4 % and 2519/3844 = 65.9 %, respectively (Fig. 2). Among the true DEGs identified by our method, 28 were detected by our method exclusively. A close examination shows that the differential expression levels for these genes are moderate on both platforms; however, they are consistent across platforms (Fig. 3). Because our method not only takes account of the significance on individual platforms but also the consistency across platforms, these genes are ranked higher than the genes that are more significant in a single platform but inconsistent across platforms.

**Fig. 4:**
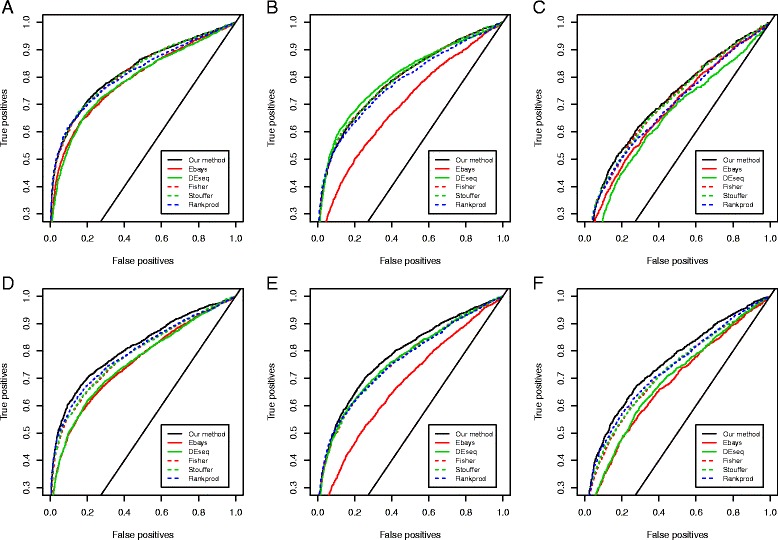
Comparison of discriminative power in the real data-based simulation study. Figures show the percentage of correct and incorrect calls at various thresholds for six simulation settings. The AUCs are shown in Additional file 1: Table S2. **a**. 20 % genes are up-regulated and 20 % genes are down-regulated. Both platforms have high data quality. **b**. 20 % genes are up-regulated and 20 % genes are down-regulated. One platform has high data quality and the other platform has low data quality. **c**. 20 % genes are up-regulated and 20 % genes are down-regulated. Both platforms have low data quality. **d**. 10 % genes are up-regulated and 30 % genes are down-regulated. Both platforms have high data quality. **e**. 10 % genes are up-regulated and 30 % genes are down-regulated. One platform has high data quality and the other platform has low data quality. **f**. 10 % genes are up-regulated and 30 % genes are down-regulated. Both platforms have low data quality

As shown in Fig. 4 and Additional file 1: Table S2, when the data consists of similar proportions of up- and down-regulated genes (Fig. 4a-c), our method and the other meta-analysis methods perform similarly. However, when the proportions of up- and down-regulated genes are considerably different (Fig. 4d-f), our method outperforms all the other methods and shows the highest area-under-the-curve (AUC) in the ROC curve (D: AUC_our_ = 0.812 vs AUC_other_ = 0.756-0.796; E: AUC_our_ = 0.785 vs AUC_other_ = 0.675-0.766; F: AUC_our_ = 0.753 vs AUC_other_ = 0.678-0.733; see Additional file 1: Table S2 for details). This is because our method is adaptive to the data and can effectively determine its rejection region according to the shapes of tails.

One concern in data integration is that integration may be deteriorated if data from one platform has poor quality. We therefore investigate how the quality of data from individual platforms affects the identification by simulating data with different quality. We consider two scenarios: the two platforms have similar data quality, and one platform has lower quality than the other. As shown in Fig. 4b (symmetric) and e (asymmetric), when one platform has apparently lower quality than the other, integrating the two platforms does not necessarily improve the discriminative power over using only the data from the platform with better quality. However, even in this scenario, our method (AUC = 0.790 for B and 0.785 for E) still shows a discriminative power that is as competitive as the better one of the single-platform methods (AUC = 0.799 for B and 0.766 for E). When the two platforms have similar quality (Fig. 4a, c, d and f, Additional file 1: Table S2), our method shows a more obvious gain over both single-platform methods regardless if the data quality is high or low.

### Application to MAQC/SEQC project data

We apply our method to a dataset from MAQC/SEQC project [3, 12]. In this dataset, the mRNA samples were generated for universal human reference RNA (Stratagene) and human brain reference RNA (Ambion). The gene expression levels of each sample were measured by microarray and RNA-seq at multiple sites using multiple commercial platforms on multiple replicates, and were validated by qRT-PCR. Thus this dataset provides an ideal benchmark for objectively assessing the performance of our method. Here we use the microarray data generated using Affymetrix array platform at Affymetrix and the RNA-seq data generated using Illumina Hi-seq 2000 platform at BGI. Detailed information on the data can be found in [3, 12]

As the features measured by microarray data and RNA-seq data do not completely overlap, we only included features shared by both platforms in our analysis. After data processing (Additional file 1, Section 3), we obtained 14546 genes that are measured on both platforms. Among them, 836 genes were validated by Taqman PCR.

We ran DEseq and eBayes using three replicates with their default parameter settings. We then integrated the p-values from DEseq and eBayes using Fisher’s method and Stouffer’s method, and integrated the fold change of the same three replicates across platforms using our approach and RankProd. Because the significance measures from these methods are not directly comparable, we rank the genes according to the significance measure from each method, and evaluate the Spearman rank correlation between these rankings and the fold change measured by PCR for the 836 PCR-validated genes in our comparison. As shown in Table 1, our method shows the highest rank correlation (0.872) with the fold change measured by PCR, and is substantially higher than the correlations from either individual platforms or meta-analysis methods (0.714–0.765). Furthermore, we calculated the average ranks of the fold change measured by PCR for top 10, 50 and 100 differentially expressed genes identified by each method (Table 2). This quantity measures how well the significance of DEGs and the PCR fold change correspond to each other in ranks for top DEGs, which are often of primary scientific interests. A smaller value of the average rank (i.e. a top rank) indicates a better enrichment of the genes with high PCR measured fold change among the top DEGs. For the top 10 DEGs, RankProd (avg. rank = 18.2) and our method (avg. rank = 21.8) show a substantially better enrichment than all the other methods (avg. rank = 40.5–52.1). For the top 50 and 100 DEGs, our method (avg. rank = 45.7 and 69.4, respectively) shows the highest enrichment among all the methods of comparison in both cases (avg. rank of other methods = 55.2–71.3 and 89.3–99.4, respectively).

**Table 1:** Spearman correlation between PCR measured fold change /EST enrichment score and significance of differentiation for MAQC/SEQC analysis

Spearman correlation	Our method	DEseq	eBayes	Fisher	Stouffer^c^	Rankprod
Taqman PCR^a^	0.872	0.761	0.714	0.765	-	0.730
EST enrichment score^b^	0.276	0.111	0.169	0.106	0.201	0.281

^a^Correlation is calculated based on the 836 genes that are validated by Taqman PCR

^b^Correlation is calculated based on 118 brain specific genes obtained from TiGER database

^c^Correlation is not computed for Stouffer method as it generates many p-values at 0

**Table 2:** Average ranks of PCR measured fold change for top DEGs identified by different methods

DEGs	Our method	DEseq	eBayes	Fisher	Rankprod
Top 10	21.8	52.1	40.5	47.4	18.2
Top 50	45.7	57.2	71.3	55.2	56.9
Top 100	69.4	91.5	92.5	89.3	99.4

Small values represent top rankings

To evaluate the functional relevance of identified DE genes, we obtained the EST (Expressed sequence tag) enrichment score of brain preferentially expressed genes, which consists of 118 genes specifically expressed in brain, from the TiGER database (TiGER: http://bioinfo.wilmer.jhu.edu/tiger/db_tissue/est/brain-index.html). The EST enrichment score reflects the specificity of gene expression in a tissue and is expected to be correlated with the differential expression levels measured in the experimental data [36]. Here we calculated the Spearman correlation between the significance measures assigned by each method and the TiGER EST enrichment (Table 1). Though the correlation is low for all the methods (0.111–0.281), our method and RankProd show the highest correlation (our: 0.276, RankProd: 0.281, others: 0.111–0.201).

### Application to synthetic microRNA data

We next illustrate the usefulness of our method for analyzing microRNA expression, for which effective sequencing technology is still under development [35]. Here we apply our method to a dataset consisting of synthetic microRNA samples with known concentrations, measured on both microarray and sequencing platforms [25]. As the amount of RNA is known in this dataset, it enables us to compare the detected DEGs with the true DEGs. Note that there is no replicate sample in this dataset, which is in fact quite common in practice for RNA-seq studies. It thus imposes challenges for the DEG detection methods based on a single platform to produce reliable statistical inference. Integrating information across platforms nevertheless may improve the reliability of DEG detection in this situation.

This dataset consists of two samples, A and B, each of which is a mixture of synthetic RNA oligos with various concentrations, including 11 differential gene expression levels, ranging from −4 to 4. In total, there are 281 genes with log2 fold change of ±4, ±3 or ±2, 278 genes with log2 fold change of ±1 or ±0.5, and 185 genes with log2 fold change 0. Detailed experimental design can be found in [32].

Since no replicates are available, eBayes cannot be applied, and DEseq can only be used with the single-replicate option. Consequently, Fisher’s method and Stouffer’s method cannot be applied, due to lack of the p-values from eBayes. Only our method and RankProd can still be applied to combine the fold changes across platforms. Therefore, we only compare the performance of our method with RankProd but not with Fisher’s method and Stouffer’s method. To evaluate the gain over using a single platform, we also compare our method with the fold change of microarray, the fold change of RNA-seq, and the p-values from DEseq generated using the single-replicate option.

Similar to the MAQC/SEQC analysis, we use the rank correlation between the significance assigned by each method and true fold changes of RNA oligos, as an assessment of the performance. Our method shows the highest rank correlation (0.930) among all methods of comparison (DEseq: 0.888, fold change of RNA-seq: 0.888, fold change of microarray: 0.840, and RankProd: 0.894).

**Fig. 5:**
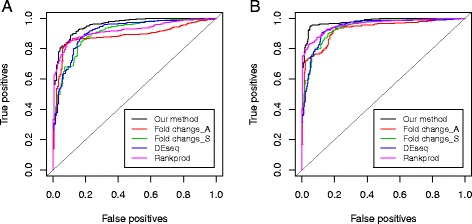
Comparison of discriminative power in the analysis of synthetic microRNA data. Figures show the percentage of correct and incorrect calls at various thresholds for two levels of classification stringency. The AUCs are shown in Additional file 1: Table S3. **a**. Genes with no fold change as true non-DEGs and the rest as true DEGs. **b**. Genes with a log2 fold change less than ±0.5 as true non-DEGs and the rest as true DEGs. Fold changes measured by microarray and RNA-seq are denoted as Fold change_A and Fold change_S, respectively

As true fold changes are known, the sensitivity and specificity of the identification of DEGs at various thresholds can be evaluated. Here we consider classification of DE and non-DE genes at two levels of stringency. One treats the genes with identical expression levels in both samples as true non-DEGs, and the rest as true DEGs; and the other treats the genes with log 2 fold change less than ±0.5 as true non-DEGs, and the rest as true DEGs. As shown in the ROC curve (Fig. 5), our method has the highest area under the curve (AUC = 0.957 for cutoff = 0; AUC = 0.978 for cutoff = ±0.5) among all methods of comparison in both cases (Other methods: AUC = 0.885–0.919 for cutoff = 0 and AUC = 0.927–0.958 for cutoff = ±0.5, see Additional file 1: Table S3).

## Conclusions

In this paper, we present a semi-parametric statistical model for integrating gene expression profiles across studies. This method has several desirable properties as a data integration method. First, it is rank-based, thus is robust to noise in the data and offers a natural way to overcome the heterogeneity across datasets, especially datasets across different platforms. Second, it builds in a strong preference for common directionality of significance across samples, thus allowing genes that have moderate differential expression levels, but are consistent across studies, to be effectively identified. Third, comparing with the commonly-used nonparametric meta-analysis methods, it is adaptive and can reflect the asymmetry in its rejection regions, when the proportions of up- and down-regulation are asymmetric.

As shown in our application to integrate gene expression levels measured on microarray and RNA-seq platforms, our method effectively improved the biological relevance of the identified DEGs. Therefore, this method provides researchers a tool that can take advantage of the gene expression data on different platforms. Though we only illustrate the integration across microarray and RNA-seq platforms, this method is generic and can be applied to integrate rank lists from different sources in other high-throughput settings. The R code for this method is available upon request.

## Additional file

Additional file 1: **Supplementary materials.** Section 1: Estimation algorithm for our model. Section 2: Simulation procedure for the real data-based simulation study. Section 3: Pre-processing procedure for MAQC/SEQC data. Section 4: Extension of our model to the case of more than two samples. Section 5: Tables S1-S3. Section 6: Figure S1-S2. (PDF 415 kb)

## Abbreviations

DEG: differentially expressed genes
