# Supplementary material for: A semi-parametric statistical model for integrating gene expression profiles across different platforms
Source: BMC Bioinformatics. 2016 Jan 11;17(Suppl 1):5. doi: 10.1186/s12859-015-0847-y (PMC4895261; doi:10.1186/s12859-015-0847-y)
Supplement: Supplementary file 1 — Supplementary materials. Section 1: Estimation algorithm for our model. Section 2: Simulation procedure for the real data-based simulation study. Section 3: Pre-processing procedure for MAQC/SEQC data. Section 4: Extension of our model to the case of more than two samples. Section 5: Tables S1-S3. Section 6: Figure S1-S2. (PDF 415 kb) [file 12859_2015_847_MOESM1_ESM.pdf]

Supplementary Materials

By Yafei Lyu and Qunhua Li

1 Estimation algorithm for the copula mixture model

1.1 Iterative procedure to esimate the parameter

Here we describe the iterative procedure to esimate the parameter $\theta = \left(\pi_0, \pi_1, \pi_2, \mu_1, \sigma_1^2, \rho_1, \mu_2, \sigma_2^2, \rho_2\right)$ in detail.

(a) Compute the empirical marginal CDF $\hat{F}_j(x_{i,j}) = \frac{r_{i,j}}{n}$ where $r_{i,j}$ is the rank of $x_{i,j}$ on the platform j and n is the sample size.

(b) Rescale $\hat{F}_j(x_{i,j})$ by $u_{i,j} \equiv \frac{n}{n+1}\hat{F}(x_{i,j})$ to avoid potential unboundedness of $G^{-1}(u_{i,j})$ if $u_{i,j}$'s tend to one.

(c) Initialize $\theta = \theta_0$.

(d) Compute pseudo-data $z_{i,j} = G^{-1}(u_{i,j} \mid \theta)$. As G^{-1} does not have a closed form, G is first computed on a grid of 1000 points for $u \in [\min(-3, \mu_2 - 3), \max(3, \mu_1 + 3)]$, then $z_{i,j}$ is obtained by linear interpolation on the grid.

(e) Run EM to maximize the log-likelihood of pseudo-data,

$$l(\theta) = \sum_{i=1}^{n} \left[\log \left\{ \pi_0 h_0(z_{i,1}, z_{i,2}; 0, 1, 0) + \sum_{k=1}^{2} \pi_k h_k(z_{i,1}, z_{i,2}; \mu_k, \sigma_k^2, \rho_k) \right\} \right]$$

to get $\theta^{(t)} = \arg\max_{\theta} l(\theta)$. The E-step and M-step are described below.

(f) Set $\theta = \theta^{(t)}$ and go to step (e) until covergence.

1.2 EM algorithm for maximizing the log-likelihood of pseudo data

Here we decrible the EM algorithm in step (f) above. To Proceed, we denote K_i as the latent variables, then the complete log-likelihood for the augmented pseudo data $Y_i \equiv (Z_i, K_i)$ is

$$\begin{aligned} l_c(\theta) &= \sum_{i=1}^{n} \Big[I(K_i = 0) \left\{ \log \pi_0 + \log h_0(z_{i,1}, z_{i,2}; 0, 1, 0) \right\} \\ &\quad + \sum_{k=1}^{2} I(K_i = k) \left\{ \log \pi_k + \log h_k(z_{i,1}, z_{i,2}; \mu_k, \sigma_k^2, \rho_k) \right\} \Big] \end{aligned}$$

where $I(\cdot)$ is the indicator function. Denote $\tau_{i,k} = P(K_i = k), \quad k = 0, 1, 2.$

(a) E-step:

Conditional on observations and current value $\theta^{(t)}$, the expected log-likelihood function is:

$$
\begin{aligned}
Q(\theta \mid \theta^{(t)}) &= \mathrm{E}\left[l_c(\theta)|Z_i, \theta^{(t)}\right] \\
&= \sum_{i=1}^{n} \Bigg[\tau_{i,0}^{(t+1)} \left\{\log \pi_0 + \log h_0(z_{i,1}, z_{i,2}; 0, 1, 0)\right\} \\
&\quad + \sum_{k=1}^{2} \tau_{i,k}^{(t+1)} \left\{\log \pi_k + \log h_k(z_{i,1}, z_{i,2}; \mu_k, \sigma_k^2, \rho_k)\right\} \Bigg]
\end{aligned}
$$

Then,

$$
\begin{aligned}
\tau_{i,0}^{(t+1)} &= \frac{P(K_i = 0, z_i \mid \theta^{(t)})}{P(z_i \mid \theta^{(t)})} \\
&= \frac{\pi_0^{(t)} h_0(z_{i,1}, z_{i,2}; 0, 1, 0)}{\pi_0^{(t)} h_0(z_{i,1}, z_{i,2}) + \sum_{k=1}^{2} \pi_k^{(t)} h_k(z_{i,1}, z_{i,2}; \mu_k^{(t)}, \sigma_k^{2(t)}, \rho_k^{(t)})} \\
\tau_{i,k}^{(t+1)} &= \frac{P(K_i = k, z_i \mid \theta^{(t)})}{P(z_i \mid \theta^{(t)})} \\
&= \frac{\pi_k^{(t)} h_k(z_{i,1}, z_{i,2}; \mu_k^{(t)}, \sigma_k^{2(t)}, \rho_k^{(t)})}{\pi_0^{(t)} h_0(z_{i,1}, z_{i,2}; 0, 1, 0) + \sum_{k=1}^{2} \pi_k^{(t)} h_k(z_{i,1}, z_{i,2}; \mu_k^{(t)}, \sigma_k^{2(t)}, \rho_k^{(t)})},
\end{aligned}
$$

for $k = 1, 2$.

(b) M-step:
By setting $\frac{\partial Q(\theta|\theta^{(t)})}{\partial \pi_k} = 0$ for $k = 0, 1, 2$, we can obtain

$$
\pi_k^{(t+1)} = \frac{1}{n} \sum_{i=1}^{n} \tau_{i,k}^{(t+1)}.
$$

Note that the first term of $Q(\theta|\theta^{(t)})$ is irrelevant to $(\mu_k, \sigma_k^2, \rho_k)$ for $k = 1, 2$. Thus, to update $(\mu_k, \sigma_k^2, \rho_k)$, we only need to maximize the first term of $Q(\theta|\theta^{(t+1)})$, which is denoted as $Q_1(\theta|\theta^{(t)})$:

$$
\begin{aligned}
& Q_1(\theta|\theta^{(t)}) \\
= & \sum_{i=1}^{n} \tau_{i,0}^{(t+1)} \left[\log\left(\frac{1}{2\pi}\right) - \frac{1}{2}\left(z_{i,1}^2 + z_{i,2}^2\right)\right] \\
& + \sum_{k=1}^{2} \sum_{i=1}^{n} \tau_{i,k}^{(t+1)} \left[\log\left(\frac{1}{2\pi\sigma_k^2\sqrt{1-\rho_k^2}}\right) - \frac{(z_{i,1}-\mu_k)^2 - 2\rho_k(z_{i,1}-\mu_k)(z_{i,2}-\mu_k) + (z_{i,2}-\mu_k)^2}{2(1-\rho_k^2)\sigma_k^2}\right]
\end{aligned}
$$

Taking derivatives w.r.t each term, we have the following:

$$
\frac{\partial Q_1(\theta|\theta^{(t)})}{\partial \mu_1} = \sum_{i=1}^{n} \frac{\tau_{i,1}^{(t+1)}}{2(1-\rho_1^2)} \cdot \frac{2(1-\rho_1)(z_{i,1} + z_{i,2} - 2\mu_1)}{\sigma_1^2}
$$

Set it equal to 0, we have:

$$
\mu_1^{(t+1)} = \frac{\sum_{i=1}^{n} \tau_{i,1}^{(t+1)}(z_{i,1} + z_{i,2})}{2\sum_{i=1}^{n} \tau_{i,1}^{(t+1)}}
$$

By symmetry,

$$
\mu_2^{(t+1)} = \frac{\sum_{i=1}^{n} \tau_{i,2}^{(t+1)}(z_{i,1} + z_{i,2})}{2\sum_{i=1}^{n} \tau_{i,2}^{(t+1)}}
$$

Similarly,

$$\begin{aligned}
\frac{\partial Q_1(\theta|\theta^t)}{\partial \sigma_1^2} &= \sum_{i=1}^{n} \tau_{i,1}^{(t+1)} \times \\
&\left[-\frac{1}{\sigma_1^2} + \frac{(z_{i,1}-\mu_1)^2 - 2\rho_1(z_{i,1}-\mu_1)(z_{i,2}-\mu_1) + (z_{i,2}-\mu_1)^2}{2\sigma_1^4(1-\rho_1^2)}\right] \quad (1)\\
\frac{\partial Q_1(\theta|\theta^t)}{\partial \rho_1} &= \sum_{i=1}^{n} \tau_{i,1}^{(t+1)} \times \\
&\left[\frac{\rho_1}{1-\rho_1^2} - \frac{\rho_1}{(1-\rho_1^2)^2} \cdot \frac{(z_{i,1}-\mu_1)^2 - (\frac{1}{\rho_1}+\rho_1)(z_{i,1}-\mu_1)(z_{i,2}-\mu_1) + (z_{i,2}-\mu_1)^2}{\sigma_1^2}\right]. \quad (2)
\end{aligned}$$

Solving (1) and (2) together,

$$\begin{aligned}
(\sigma_1^2)^{(t+1)} &= \frac{\sum_{i=1}^{n} \tau_{i,1}^{(t+1)} \left[(z_{i,1}-\mu_1)^2 + (z_{i,2}-\mu_1)^2\right]}{2\sum_{i=1}^{n} \tau_{i,1}^{(t+1)}} \\
\rho_1^{(t+1)} &= \frac{2\sum_{i=1}^{n} \tau_{i,1}^{(t+1)} (z_{i,1}-\mu_1)(z_{i,2}-\mu_1)}{\sum_{i=1}^{n} \tau_{i,1}^{(t+1)} \left[(z_{i,1}-\mu_1)^2 + (z_{i,2}-\mu_1)^2\right]}.
\end{aligned}$$

By symmetry,

$$\begin{aligned}
(\sigma_2^2)^{(t+1)} &= \frac{\sum_{i=1}^{n} \tau_{i,2}^{(t+1)} \left[(z_{i,1}-\mu_2)^2 + (z_{i,2}-\mu_2)^2\right]}{2\sum_{i=1}^{n} \tau_{i,2}^{(t+1)}} \\
\rho_2^{(t+1)} &= \frac{2\sum_{i=1}^{n} \tau_{i,2}^{(t+1)} (z_{i,1}-\mu_2)(z_{i,2}-\mu_2)}{\sum_{i=1}^{n} \tau_{i,2}^{(t+1)} \left[(z_{i,1}-\mu_2)^2 + (z_{i,2}-\mu_2)^2\right]}.
\end{aligned}$$

As shown in Figure 1, our method is well-calibrated in all the scenarios, even though our model assumption is violated. In addition, our method also shows the highest discriminative power among all the methods of comparison.

2 Real data-based simulation study

2.1 Simulation of RNA-seq data

The RNA-seq data was simulated from a negative binomial model as previously described by Kvam[1]. The simulation procedure is as follows.

(a) Simulation of mean gene expression levels

  i. We assume that the expression level of RNA-seq data follows a Gamma distribution, $Gamma(k, \theta)$. We estimate k and θ by fitting the RNA-seq data of liver and kidney samples in Marioni et al[2].

  ii. Then, we generate the mean expression level of a gene j by $a_j \sim Gamma(k, \theta)$.

(b) Simulation of fold changes

  i. We assume the log2 fold change follows a three-component Gaussian mixture distribution with $\mu_0 = 0$ for non-DE genes, $\mu_1 > 0$ for up-regulated genes and $\mu_2 < 0$ for down-regulated genes. We fit this model using the a log2 fold change between kidney and liver.

  ii. We then simulate the log2 fold change for gene j, b_j, using the three-component Gaussian mixture model with fitted parameters

(c) Generation of read counts

The read counts for gene j in treatment $i, (i = 0, 1)$ is simulated from a negative binomial distribution,

$$y_{ij}^S \sim NB(\mu_{ij}, \phi_j)$$

where mean $\mu_{ij} = a_j 2^{(-1)^i b_j}$ and the dispersion parameter ϕ_j is simulated from a Gamma distribution, $\phi_j \sim Gamma(0.85, 0.5)$, following Hardcastle and Kelly[3].

2.2 Simulation of microarray data

The simulation of microarray data follows a method in Xiao et al [4] as follows.

(a) Estimation of mean expression levels

The microarray intensity for gene j is modeled according to Rocke and Durbin [5]:

$$y_j = u + a_j \exp(\eta) + \epsilon,$$

where a_j is the intensity measured on the microarray, u is background noise, η and ϵ are error terms that are normally distributed.

To estimate a_j, one may apply a variance stablizing data transformation [6]:

$$a_j = \ln(y_j - u + \sqrt{(y_j - u)^2 + sd(\epsilon)/sd(\eta)}) \tag{3}$$

According to this model, when a_j is small, $y_j = u + \epsilon$. Since u is a constant, $sd(\epsilon) = sd(y_j)$. We used the bottom 1% data to estimate $sd(\epsilon)$. We estimate u using the mean expression of raw data minus mean expression of data after background correction, where the background correction was done by using RMA method [7]. When a_j is sufficiently large, $\log(y_j) = \log(a_j) + \eta$. So we calculate $sd(\eta)$ using genes among the top 0.1% within each treatment. The distribution of a_j then can be constructed using the estimated values through (3).

(b) Estimation of log2 fold changes

The log2 fold change is estimated from the kidney/liver microarray data in Marioni et al.[4] by assuming a three-component mixture model with $\mu_0 = 0$ for non-DE genes.

(c) Simulation of mean expression levels and log2 fold changes

For each gene, the mean expression level a_j is drawn from the distribution estimated in step 1, and the log2 fold change, b_j, is drawn from the three-component mixture model estimated from real data in step (b). Then simulated expression level is $y_{ij}^M = a_j 2^{(-1)^i b_j}$

2.3 Coupling of RNA-seq and microarray data

To reflect the correspondence between RNA-seq and microarray data measured from the same sample, we couple the RNA-seq and microarray data in the following way. For gene j, we first randomly generate percentiles $q_j^a \sim Unif(0, 1)$ and $q_j^b \sim Unif(0, 1)$ for a_j and b_j, respectively, and then simulate the RNA-seq and microarray according to 3.1 and 3.2, respectively, using the same a_j and b_j.

2.4 Procedure for adjusting data quality in simulation

This section describes the procedure and parameter settings for simulating RNA-seq and microarray data with different qualities.

For RNA-seq, we adjust the quality of data by altering the dispersion parameter ϕ_j in the negative binomial distribution. A larger ϕ_j will introduce higher level of randomness in the final expression level, reducing the quality of data. In our simulation, we draw ϕ_j from $Gamma(0.85, 0.5)$ for high-quality data, following the parameter setting in [3], and draw ϕ_j from $Gamma(1.75, 3)$ and $Gamma(1.2, 3)$ for low quality data with equal tails and unequal tails, respectively.

For microarray data, we adjust the quality of data by multiplying a random perturbation factor to the gene expression level simulated in Section 2.2 (c). That is, the gene expression level for gene j in treatment i is $y_{ij} = a_j 2^{(-1)^i b_j} \alpha_{ij}$, where $\alpha_{ij} \sim unif(1-t, 1+t)$ is a multiplicative factor to control data quality. In our simulation, we let $t = 0.12$ and $t = 0.15$ for high quality data with equal and unequal tails, respectively, and let $t = 0.24$ and $t = 0.27$ for low quality data with equal and unequal tails, respectively.

3 Preprocessing of MAQC/SEQC data

3.1 Preprocessing of MAQC data

Microarray data from MAQC project can be downloaded from Gene Expression Omnibus with the GEO Series accession number = GSE5350. Annotation for this data was obtained from hgu133plus2.db.

The data was firstly processed using the *affy* package on Bioconductor with the default setting. The RMA method was then applied to normalize the data. For genes that correspond to multiple probes, we collapse the probes and use the mean expression level of collapsed probes as the expression level of the gene.

3.2 Preprocessing of SEQC data

RNA-seq data was obtained by loading R package *SEQC*. Then the data was normalized by using DEseq package with the default setting. The read counts were summed over all 20 replicates for each gene symbol. The symbols with total read count less than 30 are removed from analysis. Only the gene symbols that are shared by both RNA-seq data and microarray data are kept for further analysis.

4 Extension of our model to the case of more than two samples

When more than 2 ($m > 2$) samples are integrated, the copula mixture model can be described as follows.

Let $K_i = k \sim Bernoulli(\pi_k)$, k=0, 1, 2, and

$$\begin{pmatrix} \sigma_k^2 & \rho_k\sigma_k^2 & \cdots & \rho_k\sigma_k^2 \\ \rho_k\sigma_k^2 & \sigma_k^2 & \cdots & \rho_k\sigma_k^2 \\ \vdots & \vdots & \ddots & \vdots \\ \rho_k\sigma_k^2 & \rho_k\sigma_k^2 & \cdots & \sigma_k^2 \end{pmatrix},$$

where $\mu_0 = 0$, $\mu_1 > 0$, $\mu_2 < 0$, $\rho_0 = 0$, $0 < \rho_1 \leq 1$, $0 < \rho_2 \leq 1$.

Let

$$u_{i,j} \equiv G(z_{i,j}) = \sum_{k=0}^{2} \pi_k \Phi(\frac{z_{i,j} - \mu_k}{\sigma_k})$$

where $j = 1, \ldots, m$. Our actual observations are

$$x_{i,j} = F_j^{-1}(u_{i,j})$$

5 Supplementary tables

Table 1: Parameter estimation for the simulation with violation of model assumptions

Parameter	S1		S2		S3		S4	
	True	Estimated	True	Estimated	True	Estimated	True	Estimated
π_0	0.50	0.511 (0.010)	0.50	0.510 (0.010)	0.90	0.899 (0.003)	0.50	0.504 (0.009)
π_1	0.25	0.242 (0.005)	0.35	0.343 (0.007)	0.05	0.050 (0.002)	0.25	0.248 (0.004)
π_2	0.25	0.245 (0.006)	0.15	0.145 (0.005)	0.05	0.050 (0.002)	0.25	0.247 (0.005)
μ_1	0.58–1.58	1.087 (0.032)	0.58–1.58	1.097 (0.014)	0.58–1.58	1.107 (0.036)	0.58–1.58	1.089 (0.012)
μ_2	-1.58– -0.58	-1.095 (0.016)	-1.58 – -0.58	-1.098 (0.036)	-1.58– -0.58	-1.114 (0.037)	-1.58– -0.58	-1.088 (0.011)
σ_1	0.80	0.807 (0.019)	0.80	0.801 (0.011)	0.80	0.815 (0.372)	0.80	0.813 (0.010)
σ_2	0.80	0.801 (0.017)	0.80	0.815 (0.019)	0.80	0.827 (0.335)	0.80	0.812 (0.010)
ρ_1	0.80–0.88	0.852 (0.014)	0.80–0.88	0.861 (0.015)	0.80–0.88	0.844 (0.024)	0.55–0.65	0.643 (0.017)
ρ_2	0.80–0.88	0.855 (0.016)	0.80–0.88	0.838 (0.024)	0.80–0.88	0.853 (0.024)	0.55–0.65	0.643 (0.022)

Four simulation scenarios:

S1: data with same proportion of up- and down-regulated DEGs

S2: data with different proportions of up- and down-regulated DEGs

S3: data with a small proportion of DEGs

S4: data with low inter-platform consistency.

Table 2: AUC for the real data-based simulation study

Cases	Tails[1]	Data quality[2]	Our method	eBayes	DEseq	Fisher	Stouffer	RankProd
(A)		H H	**0.823**	0.790	0.787	0.820	0.821	0.814
(B)	Equal	H L	0.790	0.695	**0.799**	0.791	0.792	0.779
(C)		L L	**0.714**	0.685	0.649	0.707	0.707	0.690
(D)		H H	**0.812**	0.756	0.757	0.787	0.789	0.796
(E)	Unequal	H L	**0.785**	0.675	0.766	0.765	0.766	0.764
(F)		L L	**0.753**	0.678	0.687	0.722	0.724	0.733

The highest AUC in each case is shown in bold face.

[1] Scenarios with different proportions of up- and down-regulated genes

- Equal: there are 20% up-regulated genes, 20% down-regulated genes and 60% non-DEGs.
- Unequal: there are 30% up-regulated genes, 10% down-regulated genes and 60% non-DEGs.

[2] Scenarios with different data quality

- H H: Both RNA-seq and microarray data have high quality.
- H L: RNA-seq data has high quality while microarray data has low quality.
- L L: Both RNA-seq and microarray data have low quality.

Table 3: AUC for the synthetic microRNA data

Cutoff[1]	Our method	RNA-seq fold change	Microarray fold change	DEseq	RankProd
0	**0.957**	0.906	0.885	0.913	0.919
±0.5	**0.978**	0.938	0.927	0.942	0.958

The highest AUC in each case is shown in bold face.

[1] AUC was calculated for two levels of classification stringency.

- Cutoff=0: Genes with no fold change as true non-DEGs and the rest as true DEGs
- Cutoff=±0.5: Genes with a log2 fold change less than ±0.5 as true non-DEGs and the rest as true DEGs and the rest as true DEGs

6 Supplementary Figures

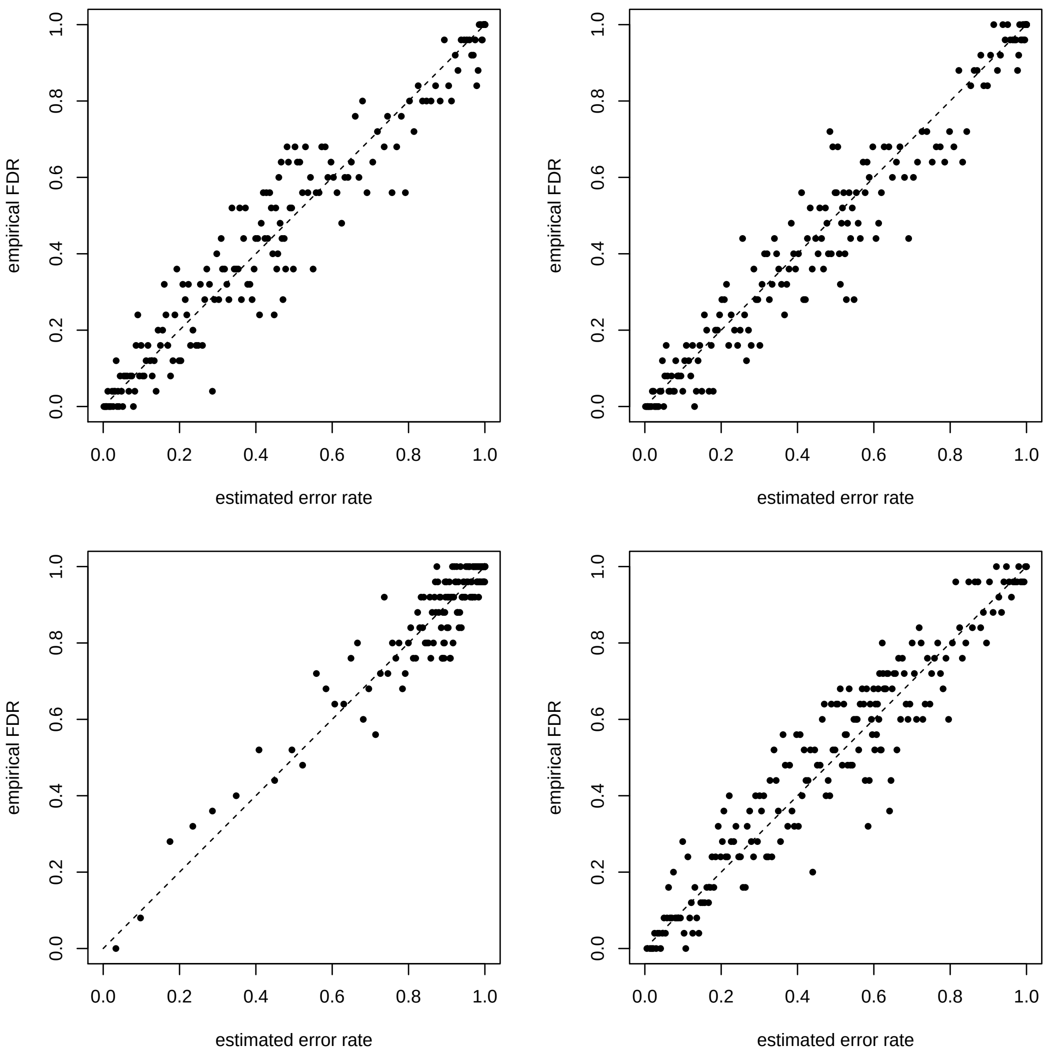

Figure 1: Calibration of our method in the simulation with violation of model assumption. The estimated error rate (x-axis) is compared with the actual frequency of false identifications (y-axis) in four simulation settings. (A) S1: data with same proportion of up- and down-regulated DEGs, (B) S2: data with different proportions of up- and down-regulated DEGs, (C) S3: data with a small proportion of DEGs, (D) S4: data with low inter-platform consistency. Parameters for each setting are shown in Supplementary Table 1. In all settings, our method shows a good calibration.

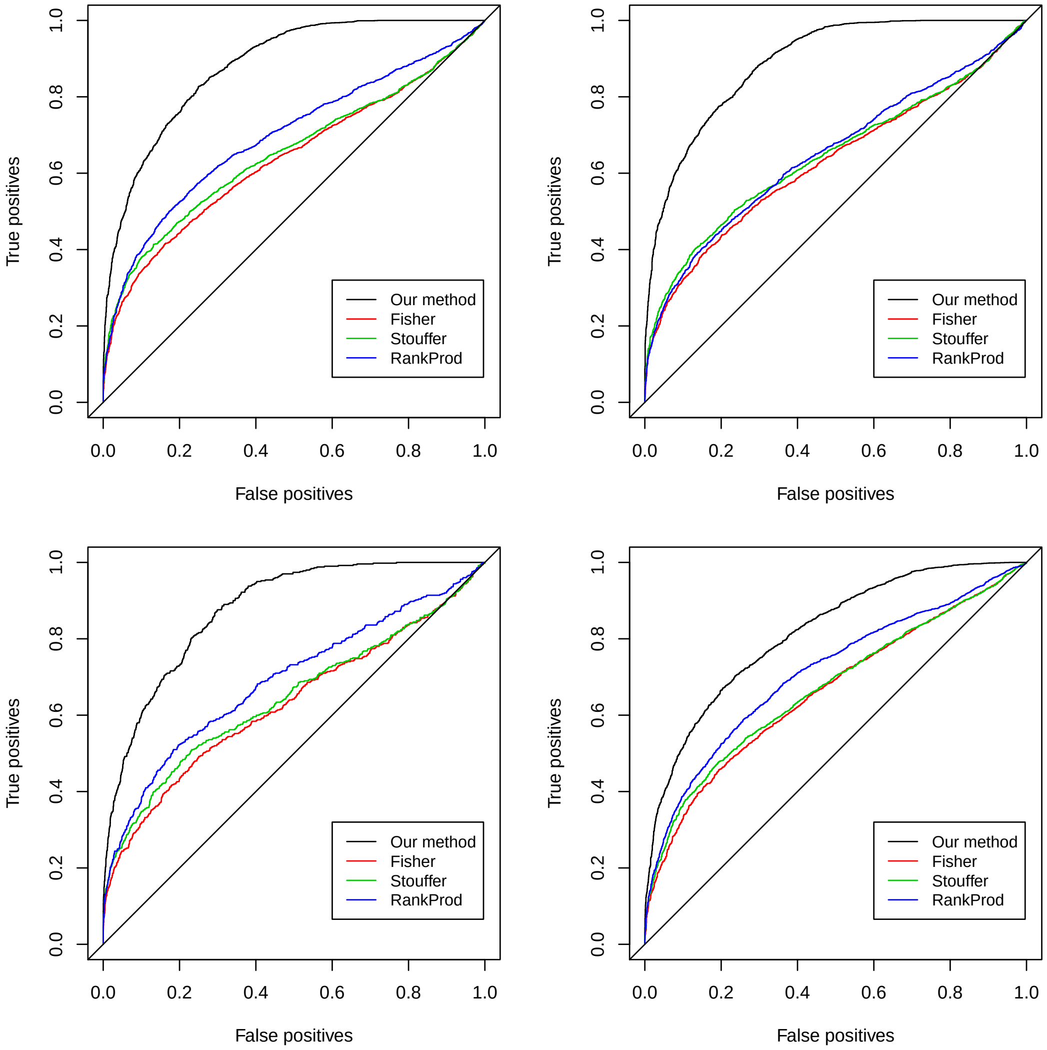

Figure 2: Comparison of discriminative power in the simulation with violation of model assumption. The percentage of correct and incorrect calls at various thresholds for our method, Fisher's method, Stouffer's method and RankProd in four simulation settings. (A) S1: data with same proportion of up- and down-regulated DEGs, (B) S2: data with different proportions of up- and down-regulated DEGs, (C) S3: data with a small proportion of DEGs, (D) S4: data with low inter-platform consistency. Parameters for each setting are shown in Supplementary Table 1. In all settings, our method outperforms all the other methods.

7 References

[1] Kvam VM, Liu P, Si Y. A comparison of statistical methods for detecting differentially expressed genes from RNA-seq data. American journal of botany 2012, 99(2):248-256.

[2] Marioni JC, Mason CE, Mane SM, Stephens M, Gilad Y. RNA-seq: an assessment of technical reproducibility and comparison with gene expression arrays. Genome research 2008, 18(9):1509-1517.

[3] Hardcastle TJ, Kelly KA. baySeq: empirical Bayesian methods for identifying differential expression in sequence count data. BMC bioinformatics 2010, 11(1):422.

[4] Xu X, Zhang Y, Williams J, Antoniou E, McCombie WR, Wu S, Zhu W, Davidson NO, Denoya P, Li E. Parallel comparison of Illumina RNA-Seq and Affymetrix microarray platforms on transcriptomic profiles generated from 5-aza-deoxy-cytidine treated HT-29 colon cancer cells and simulated datasets. BMC bioinformatics 2013, 14(Suppl 9):S1.

[5] Rocke DM, Durbin B. A model for measurement error for gene expression arrays. Journal of computational biology 2001, 8(6):557-569.

[6] Anders S, Huber W. Differential expression analysis for sequence count data. Genome biol 2010, 11(10):R106.

[7] Irizarry RA, Hobbs B, Collin F, Beazer-Barclay YD, Antonellis KJ, Scherf U, Speed TP. Exploration, normalization, and summaries of high density oligonucleotide array probe level data. Biostatistics 2003, 4(2):249-264.
